# *Psoralea margaretiflora* (Psoraleeae, Fabaceae): A new species from the Sneeuberg Centre of Floristic Endemism, Eastern Cape, South Africa

**DOI:** 10.3897/phytokeys.5.1585

**Published:** 2011-07-27

**Authors:** Charles H. Stirton, V. Ralph Clark, Nigel P. Barker, A. Muthama Muasya

**Affiliations:** 1 Bolus Herbarium, Botany Department, University of Cape Town, Rondebosch, 7700, South Africa; 2 Department of Botany, Rhodes University, Grahamstown, 6140, South Africa

**Keywords:** Eastern Cape, endemic, Fabaceae, Great Escarpment, Leguminosae, new species, Psoraleeae, *Psoralea*, Sneeuberg Centre, South Africa, taxonomy

## Abstract

A new species of *Psoralea* is described. *Psoralea margaretiflora* C.H. Stirton & V.R. Clark is endemic to the Sneeuberg Centre of Floristic Endemism, Eastern Cape, South Africa. This resprouter is characterised by its small greenish-white flowers with a small trifid purple nectar patch and translucent veins; 5(–7)-pinnate leaflets; multi-branching erect short seasonal flowering shoots; and tall habit of many stiff bare stems with the seasonal shoots massed at the apex. It is most similar to *Psoralea oligophylla* Eckl. & Zeyh., a widespread species found in the Eastern Cape. The reseeder *Psoralea oligophylla* differs in its lax virgate spreading habit with numerous long glaucous seasonal shoots; single stem, 1(–3)- glaucous leaflets; more numerous white flowers; and standard petals with a purple ring surrounding a bright yellow nectar patch.

## Introduction

There are eight species of *Psoralea* L. in eastern and south-eastern South Africa.Although well-collected and well-represented in herbaria, the known species names are mostly misapplied. These *Psoralea* species are quite variable and are now in much need of revision following Forbes’ (1930) original treatment. *Psoralea margaretiflora* – the new species described here – was not seen by Miss Helena Forbes, and occurs in the Sneeuberg, in the Eastern Cape Province of South Africa. The Sneeuberg was designated the Sneeuberg Centre of Floristic Endemism by Clark et al. (2009), and *Psoralea margaretiflora* is the latest addition to a suite of ca. 28 endemics (Goldblatt and Manning 2007, Clark et al. 2009, Nordenstam et al. 2009) known from there.

## Species treatment

### Psoralea margaretiflora

C.H. Stirton & V.R. Clark sp. nov.

urn:lsid:ipni.org:names:77112772-1

http://species-id.net/wiki/Psoralea_margaretiflora

Figs 1 2 Plate 1 

#### Latin.

Psoralea oligophylla *Eckl. & Zeyh. affinis, sed floribus parvis viridi-albis, cum macula nectarifera purpurea trifida, venis petalorum translucentibus; foliolis 5(–7)-pinnatis; brachyblastis floriferis vernalis brevibus erectis ramosis; habitu repullulanti caulibus nudis rigidis elatis multis, brachyblastis ad apicem acervulatis differt.*

#### Type.

South Africa: Eastern Cape: Graaff-Reinet Dist., Farm 360, Petersburg, Asante Sana Private Game Reserve, Suurkloof in the Sneeuberg mountains, 32°16'20"S, 25°00'05"E  (3225AC), Afro-montane shrubland on lower slopes, 1 400 m, March 2008, *V.R. Clark & I. Crause* 4 (holotype: GRA!; isotypes: BOL! K! NBG! NSW! PRE! S!).

#### Description.

*Erect resprouter*, up to 2 m. *Stems* 1–30, bare except for seasonal shoots in the upper axils; greyish-brown, covered in white storied lenticels; young seasonal shoots bright green, glabrous, glandular; shoots produced seasonally on old stems, leafy along entire length. *Stipules* 2–3 (4) mm long, rigid, triangular, semi-patent; longer, green and arching on water shoots; rapidly senescent on flowering shoots. *Leaves* 7-foliolate at base of each seasonal shoot, 5-foliolate thereafter, glabrous. Leaf size variable, larger (48–55 mm long, 48–60 mm wide) on water shoots from the rootstock; petiole 2–3 (17) mm long. *Leaflets* of variable length in a leaf; basal pair longest (25–33 mm long), mid-pair shortest (19–26 mm long), and terminal leaflet second longest (18–31 mm long); all 1.0–1.3 mm wide; glabrous, dark green; apex acuminate, base rounded. *Peduncles* (10) 15–17 mm long, terminated by a tri-toothed cupulum; lower tooth longest, acuminate, upper two teeth fused for half their length; yellowish, rapidly senescent, 1.0–1.2 mm long; pedicels 1–2 mm long. *Flowers* 10–12 mm long, greenish white, borne 1–5 in leaf axils along flowering shoot. *Standard* broadly elliptic, 10 mm long, 8 mm wide, claw 3 mm long; greenish-white, nectar flash purple, trifid above the strongly developed auricles. *Calyx* 5–6 mm long, pale green, glabrous on outside, finely black-haired on inner face of teeth, tube glabrous; teeth and tube equal, teeth triangular, all 3 mm long, carinal tooth cucullate at apex; ribbed, glandular. *Wing petals* 9–10 mm long, 4 mm wide, claw 3 mm long; locked into keel but not fused; longer than the keel; petal sculpturing present, upper basal, comprising 7–8 transcostal parallel lamellae. Keel petals 6 mm long, 3 mm wide, claw 5 mm long, apex deep purple. *Androecium* 9 mm long; tenth stamen free; sheath split adaxially, fenestrate. *Pistil* 9 mm long; ovary 1.5 mm long, stipitate, glabrous; thickened at point of flexure, height of curvature 2 mm, erect, penicillate. *Fruits* and seeds unknown.

**Figure 1.**
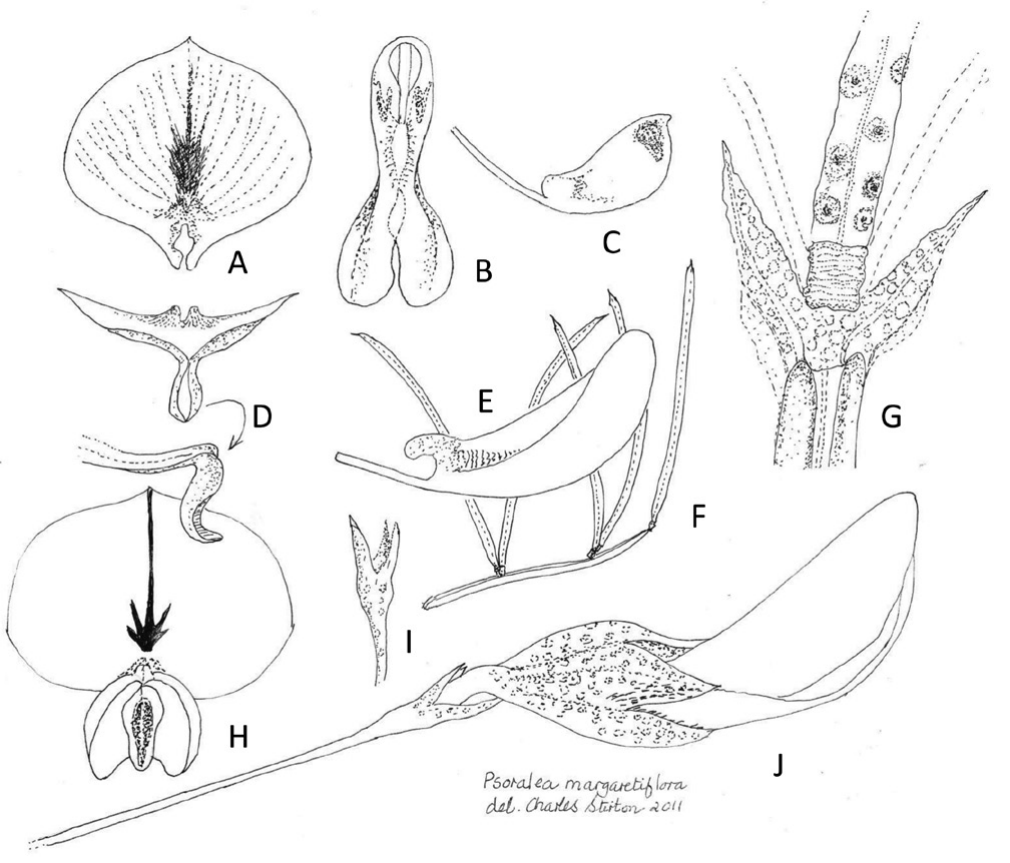
*Psoralea margaretiflora* C.H. Stirton & V.R. Clark **A** Standard petal - abaxial view (x9) **B** wing and keel petals viewed abaxially showing androecial fenestration at the base (x9) **C** keel petal (x9) **D **standard petal: abaxial and side views in reflexed position, showing raised callosities at the base above the claw (x9) **E** wing petal (x11) **F** leaf from seasonal shoot (x3.5) **G** paired stipules fused across their base (x30) **H** flower viewed from the front (x10) **I** trifid cupulum (x14) **J** flower subtended by a filiform peduncle terminating in a trifid cupulum of fused bracts (x14). Line drawing by Charles Stirton from voucher V.R. Clark, C.H. Stirton, & P. Weston 91 (GRA).

#### Discussion.

*Psoralea margaretiflora* is characterised by its small greenish white flowers with a small trifid purple nectar patch and translucent veins; 5(–7)- pinnate leaves; erect multi-branching short seasonal flowering shoots and tall habit of many stiff bare stems with the seasonal shoots burst-branching from the apex. It is most similar to *Psoralea oligophylla* Eckl. & Zeyh., a widespread species found in the Eastern Cape. *Psoralea oligophylla*, a reseeder, differs in its lax virgate spreading habit with numerous long glaucous seasonal shoots; single stem; 1–3- foliolate glaucous leaves; more numerous white flowers; and standard petals with a purple ring surrounding bright yellow nectar patch.

Two other species occur in the Eastern Cape that might be confused with *Psoralea margaretiflora*: *Psoralea glabra* Harv. and *Psoralea latifolia* (Harv.) C.H.Stirt. The four species can be distinguished as follows:

**Table:** 

1a	Flowers white or greenish white; standard petal without large white V-shaped nectar patch, veins either translucent or pale violet; wing petals white; calyx glabrous, glaucous or pale green; stipules triangular, flat, erect to semi-patent, not prominent or persistent on old shoots; shrubs up to 3 m tall with many rigid branchless stems bearing clusters of seasonal flowering shoots radiating either terminally from stems or along older shoots giving a broom-like appearance	2
1b	Flowers mauve or purple; standard petal with large white V-shaped nectar patch, veins purple; wing petals mauve or purple; calyx variously hairy, dark green or purplish; stipules subulate, swollen, recurved, woody and persistent on old shoots; shrubs or small trees from 3‒3.5 m tall with erect leafy stems and short seasonal shoots, not broom-like in appearance	3
2a	Robust woody shrubs up to 2 m tall with stiff erect habit; young seasonal shoots erect, upcurving, bright green; 7-foliolate at the base of seasonal shoot, 5-foliolate thereafter; flowers 2-3 per axil; nectar guide of trifid violet flashes above the strongly developed auricles, veins translucent	*Psoralea margaretiflora*
2b	Slender woody shrubs to 3m tall with lax virgate untidy spreading habit; young seasonal shoots arching, glaucous; 3-foliolate at the base of seasonal shoot, 1(3)-foliolate thereafter; flowers 1 (2) per axil; nectar guide bright yellow with very small white V-shaped apex surrounded by violet flashes, veins violet	*Psoralea oligophylla*
3a	Reseeder; flowers as long as or shorter than the subtending leaflets; leaflets dark green, flattish, 1.5‒4.5 mm wide; calyx hairy	*Psoralea latifolia*
3b	Resprouter; flowers longer than the subtending leaflets; leaflets yellowish green, somewhat canaliculate, less than 1 mm wide; calyx sparsely hairy	*Psoralea glabra*

#### Distribution and ecology.

*Psoralea margaretiflora* is abundant on the lower and mid-Escarpment slopes (1 200–1 800 m) of the Sneeuberg, Graaff-Reinet District, being concentrated on the Kamdebooberge, Koudeveldberge and Toorberg in the west, and from the Nardousberg to Aasvoëlkrans (behind Pearston) in the east. *Psoralea margaretiflora* can form dense stands, and is a typical component of riparian thicket/bush vegetation along streams, but is not restricted to such habitats. The vegetation types of which this species inhabits are difficult to classify. It occurs variously in Karoo Escarpment Grassland / “Afromontane Grassland” verging into Mountain Fynbos, and also occurs in closed *Otholobium macradenium* shrubland. The plant grows primarily on rich turf soils and colluvium associated with dolerite. On the Boschberg – the eastern, wetter end of the Sneeuberg – *Psoralea margaretiflora* is replaced by *Psoralea glabra*.

Flowering takes place between October and January but can occur as late as April.

**Figure 2.**
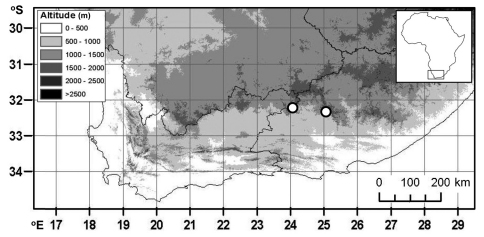
Distribution of *Psoralea margaretiflora* C.H. Stirton, & V.R. Clark.

**Plate 1.**
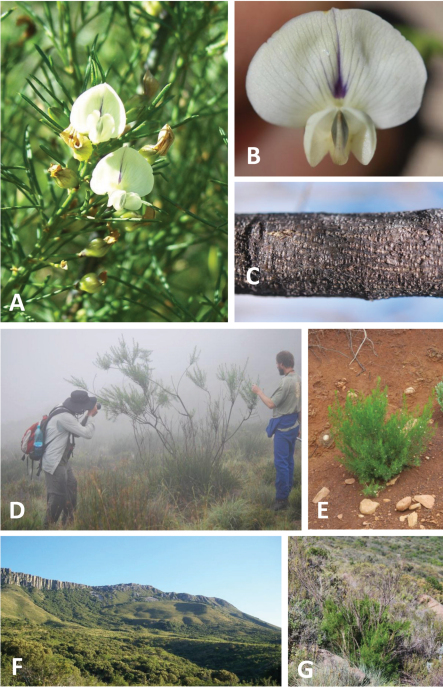
*Psoralea margaretiflora* C.H. Stirton & V.R. Clark **A** Flowers **B** flower, face view **C** main stem wood **D** mature plant **E** coppicing plant in bare ground **F** typical mountain habitat (in this case Goewermentsberg, Kamdebooberge) **G** coppicing plant in mature vegetation. Photos A & F by Ralph Clark; B – E, & G by Charles Stirton.

#### Etymology.

The plant name alludes to the appearance of its pearl white flowers as seen in early morning mountain mist and is derived from *margaritaceus* (L) = pearl-like.

#### Other specimens examined.

South Africa: Eastern Cape: Graaff-Reinet Dist.:

Petersburg, Asante Sana Private Game Reserve, Suurkloof in the Sneeuberg, ca. 32°16'S, 25°00'E  (3225AC), thicket-shrubland on lower slopes, 1 300 m, December 2005, *V.R. Clark & G. Coombs 101* (GRA! PRE!).

Farm Stockdale 387, Sneeuberg, in upper reaches of Naudeshoekspruit valley, 32°26'19"S, 25°14'56"E  (3225AD), montane riparian shrubland, 1 461 m, October 2006, *V.R. Clark & S. Ramdhani* *197* (GRA!).

Farm Onbedacht 294, eastern slopes of Koudeveldberge (Sneeuberg), 32°10'14"S, 24°03'06"E  (3224AA), Afro-montane grassland-shrubland, 1 600–1 800 m, November 2006, *V.R. Clark & T. Te Water Naudé 140* (GRA!).

Farm Buffelshoek 25, Sneeuberg, lower slopes west of Aasvoëlkrans, 32°26'6"S, 25°12'3"E  (3225AC), along watercourse in kloof in *Olea europaea* riparian thicket, 1 204 m, November 2007, *V.R. Clark & M.C. Rose* *20* (GRA!).

Farm Onbedacht 294, Koudeveldberge (Sneeuberg), 32°11'S, 24°03"E  (3224AA), afro-montane shrubland along stream, 1 600 m, December 2007, *V.R. Clark & C. Pienaar* 369 (GRA!).

Farm Oaklands 104, mid- and lower slopes of Goewermentsberg, Kamdebooberge, 32°21'15"S, 23°53'27"E  (3223BD), Afro-montane grassland-fynbos-shrubland, 1 421 m, April 2008, *V.R. Clark & I. Crause* *140* (BOL! GRA! PRE!).

Farm Oaklands 104, lower slopes of Goewermentsberg, Kamdebooberge, 32°20'58"S, 23°54'31"E  (3223BD), Afro-montane grassland-fynbos-shrubland, 1 283 m, December 2008, *V.R. Clark & C. Cloete* *1* (BOL! GRA! K! NSW! PRE!).

Plaas 96, Kamdebooberge, 32°23'35"S, 23°50'35"E  (3223BD), upper slopes of mountain in shrubland, January 2011, *V.R. Clark, C.H. Stirton & P. Weston* 2 (BOL! GRA! K! NSW! PRE!).

Farm Oaklands 104, mid-slopes of Goewermentsberg, Kamdebooberge, 32°21'15"S, 23°53'27"E  (3223BD), Afro-montane grassland—fynbos-shrubland, 1 421 m, January 2011, *V.R. Clark, C.H. Stirton & P. Weston* 91 (BOL! GRA! K! NSW! PRE!).

## Supplementary Material

XML Treatment for Psoralea margaretiflora
